# Construction and Validation of a Prognostic Risk Model for Triple-Negative Breast Cancer Based on Autophagy-Related Genes

**DOI:** 10.3389/fonc.2022.829045

**Published:** 2022-02-04

**Authors:** Cheng Yan, Qingling Liu, Ruoling Jia

**Affiliations:** ^1^ School of Pharmacy, Xinxiang University, Xinxiang, China; ^2^ Key Laboratory of Nano-Carbon Modified Film Technology of Henan Province, Xinxiang University, Xinxiang, China; ^3^ Diagnostic Laboratory of Animal Diseases, Xinxiang University, Xinxiang, China

**Keywords:** TNBC (triple-negative breast cancer), Autophagy, risk model, EIF4EBP1, prognosis

## Abstract

**Background:**

Autophagy plays an important role in triple-negative breast cancer (TNBC). However, the prognostic value of autophagy-related genes (ARGs) in TNBC remains unknown. In this study, we established a survival model to evaluate the prognosis of TNBC patients using ARGs signature.

**Methods:**

A total of 222 autophagy-related genes were downloaded from The Human Autophagy Database. The RNA-sequencing data and corresponding clinical data of TNBC were obtained from The Cancer Genome Atlas (TCGA) database. Differentially expressed autophagy-related genes (DE-ARGs) between normal samples and TNBC samples were determined by the DESeq2 package. Then, univariate Cox, least absolute shrinkage and selection operator (LASSO), and multivariate Cox regression analyses were performed. According to the LASSO regression results based on univariate Cox, we identified a prognostic signature for overall survival (OS), which was further validated by using the Gene Expression Omnibus (GEO) cohort. We also found an independent prognostic marker that can predict the clinicopathological features of TNBC. Furthermore, a nomogram was drawn to predict the survival probability of TNBC patients, which could help in clinical decision for TNBC treatment. Finally, we validated the requirement of an ARG in our model for TNBC cell survival and metastasis.

**Results:**

There are 43 DE-ARGs identified between normal and tumor samples. A risk model for OS using CDKN1A, CTSD, CTSL, EIF4EBP1, TMEM74, and VAMP3 was established based on univariate Cox regression and LASSO regression analysis. Overall survival of TNBC patients was significantly shorter in the high-risk group than in the low-risk group for both the training and validation cohorts. Using the Kaplan–Meier curves and receiver operating characteristic (ROC) curves, we demonstrated the accuracy of the prognostic model. Multivariate Cox regression analysis was used to verify risk score as an independent predictor. Subsequently, a nomogram was proposed to predict 1-, 3-, and 5-year survival for TNBC patients. The calibration curves showed great accuracy of the model for survival prediction. Finally, we found that depletion of EIF4EBP1, one of the ARGs in our model, significantly reduced cell proliferation and metastasis of TNBC cells.

**Conclusion:**

Based on six ARGs (CDKN1A, CTSD, CTSL, EIF4EBP1, TMEM74, and VAMP3), we developed a risk prediction model that can help clinical doctors effectively predict the survival status of TNBC patients. Our data suggested that EIF4EBP1 might promote the proliferation and migration in TNBC cell lines. These findings provided a novel insight into the vital role of the autophagy-related genes in TNBC and may provide new therapeutic targets for TNBC.

## Introduction

Breast cancer is the most leading diagnosed cancer among women, with the fifth highest cancer mortality worldwide in 2020 (1). Triple-negative breast cancer (TNBC) is a subtype of breast cancer, which is defined by the lack in the expression of estrogen (ER), progesterone (PR), and HER2 receptors. TNBC takes up approximately 15%–20% of total breast cancers and is the second leading cause of cancer death among women worldwide (2). TNBC is characterized by high heterogeneity, early diagnosis difficulty, rapid metastasis, poor survival, and high recurrence rate (3). Statistics show that the 5-year survival rate of TNBC patients is <40% after diagnosis (4). Although various therapeutic approaches have been proposed for TNBC, the incidences and recurrence ratios of TNBC still remain unsatisfactory, especially for developed countries (5). Tumor–nodes–metastasis (TNM) stage and molecular subtypes have been widely used in the routine diagnosis and treatment of TNBC. However, traditional markers have limited sensitivity and specificity to precisely predict prognosis and design individualized treatment in TNBC patients. Therefore, it is imperative to establish new molecular biomarkers and prognostic models to further improve the effectiveness of treatment strategies for TNBC patients.

Autophagy is an important cellular catabolic process that maintains homeostasis by eliminating aggregated proteins and damaged organelles in eukaryotic cells (6). More and more studies showed that autophagy plays a paradoxical tumor-suppressive or tumor-promoting role in different contexts and stages of progression: it prohibits tumorigenesis in the early stage but supports various tumor growth in late stage (7). Recent evidence indicated that autophagy has a high vital function in tumorigenesis and metastasis of TNBC. Indeed, several reports have indicated that TNBC tumors exhibit a higher level of autophagy than other breast cancer subtypes (8–10). Knockdown of autophagy-related genes (LC3 and Beclin-1) inhibits autophagy and significantly reduces cell proliferation, colony formation, and migration and induces apoptosis in MDA-MB-231 and BT-549 TNBC cells (11). These findings have confirmed the importance of autophagy in TNBC and suggest that ARGs may serve as prognostic markers for TNBC. To our knowledge, there is no prognosis model of ARGs in TNBC that has been constructed to predict the prognosis of TNBC patients. Therefore, a novel prognostic model with ARGs for predicting survival in TNBC is highly needed.

In this study, we analyzed in detail the transcriptome and clinical data of TNBC obtained from The Cancer Genome Atlas (TCGA) database and built an ARGs-based prognosis model using univariate Cox regression and least absolute shrinkage and selection operator (LASSO) regression analysis. Then, the proposed model was validated by the test set. Finally, we knocked down the expression level of EIF4EBP1, one of the prognosis-related genes in our model, to explore its function in TNBC. This model may provide a new reference index for the stratification of prognosis risk and treatment strategy selection of TNBC patients. Meanwhile, therapeutic targeting of EIF4EBP1 may be a potential therapeutic strategy for TNBC.

## Methods

### Data Acquisition

The Human Autophagy Database (HADb, http://www.autophagy.lu/index.html) can provide the entire set of human genes associated with autophagy (12). We collected 222 ARGs from HADB. In addition, the RNA-sequencing and corresponding clinical data of triple-negative breast cancer in TCGA were downloaded from the University of California, Santa Cruz (UCSC) XENA database (https://xena.ucsc.edu/). TNBC samples were selected using negative for “breast carcinoma estrogen receptor status,” “breast carcinoma progesterone receptor status,” and “lab procedure her2 neu *in situ* hybrid outcome type” as screening criteria. The microarray and corresponding clinical data of GSE58812 were downloaded from Gene Expression Omnibus (GEO) database (https://www.ncbi.nlm.nih.gov/geo/).

### Identification of Differentially Expressed ARGs

Differential gene expression of ARGs (DE-ARGs) in 162 normal samples and 103 TNBC samples was carried out by the DESeq2 package. We set false discovery rate (FDR) <0.05 and |log_2_ fold change (FC)| >1 as cutoff criteria to obtain DE-ARGs. Volcano plots of DE-ARGs were constructed with the OmicStudio tools (https://www.omicstudio.cn/tool); boxplots were plotted using the ggplot2 R package, Heatmaps were obtained using Morpheus (https://software.broadinstitute.org/morpheu). Protein–protein interaction (PPI) networks were generated using the STRING website (https://string-db.org).

### Functional Enrichment Analysis

We performed Gene Ontology (GO) and Kyoto Encyclopedia of Genes and Genomes (KEGG) biological process enrichment of the DE-ARGs by R statistical software including packages of “clusterProfiler”, “org.Hs.eg.db”, “enrichplot”, “ggplot2”, and “GOplot”. An adjusted p-value <0.05 was considered statistically significant. Moreover, Gene Set Enrichment Analysis (GSEA) of TCGA and GEO was conducted to reveal the signaling pathways and biological processes between high- and low-risk groups in TNBC patients (http://software.broadinstitute.org/gsea/).

### Identification of Prognostic Gene Signatures

We used the 103 TNBC samples from TCGA cohort as a training set. Univariate Cox regression analysis was performed on ARGs of train set to identify the association between the expression levels of the genes and patients’ overall survival (OS) using the “survival” package (http://bioconductor.org/packages/survival/) in R 3.6.1. The hazard ratio (HR) and p-value of each gene were calculated. Genes with p < 0.05 were screened for further analysis. Later, we further used LASSO Cox regression to reduce the number of genes and eliminate collinearity between genes. Finally, we performed multivariate Cox regression analysis based on univariate Cox regression.

### Construction and Validation of a Prognostic Model

According to the results of LASSO Cox regression, the risk scores of all samples were calculated according to the equation:


$$\text{risk score}=\sum_{j=1}^{n}\left(Coef_{j}\times Xj\right)$$

Coef refers to the regression coefficient of ARGs in LASSO Cox regression analysis, “*X*” is the expression value of the gene, and “*n*” is the number of prognostic ARGs. Using the median risk score as threshold, patients were divided into the high-risk group and low-risk group. We used the R package “survival” to assess differences in OS and obtain Kaplan–Meier survival plots. The receiver operating characteristic (ROC) curve was generated by the timeROC package to evaluate the prognostic ability of the model. Simultaneously, we used samples from GEO database as the validation set. We calculated risk scores for patients in the GEO cohort using the same formula of the train set. Then, we performed univariate and multivariate Cox regression analyses to investigate whether risk score was an independent prognostic factor for OS in TNBC patients in the train set. N, T, stage, and risk score were used as covariates. t-tests were used to test the correlation between risk score and clinicopathological factors. A p-value less than 0.05 (p < 0.05) was considered statistically significant.

### The Construction of Nomogram and Calibration Curves

Nomogram and calibration plots were generated by using the “rms” package in R software. The nomogram was used to investigate the level of consistency between the actual and predicted probabilities. Calibration plot was used to predict 1-, 3-, and 5-year survival rates.

### TNBC Cell Culture, Proliferation, Colony Formation, and Migration Assays

MDA-MB-231 and BT549 cells were cultured in Dulbecco’s modified Eagle’s medium (DMEM) medium. Two small inferring RNAs (siRNAs) were employed to knock down EIF4EBP1, and the sequences were as follows: siRNA1, 5′-GGGAGGTACCAGGATCATCTA-3′; siRNA2, 5′-GGAGGTACCAGGATCATCTAT-3′. Cell proliferation of control and EIF4EBP1 knockdown cells was determined by CCK8 and Edu. Cells transfected with control or EIF4EBP1 siRNAs were seeded in six-well plates, and colonies were measured by crystal violet staining after 15 days culture. Transwell and wound healing assays were performed and quantified using control and EIF4EBP1 knockdown cells to determine cell migration.

## Results

### Identification of DE-ARGs

**Figure 1:**
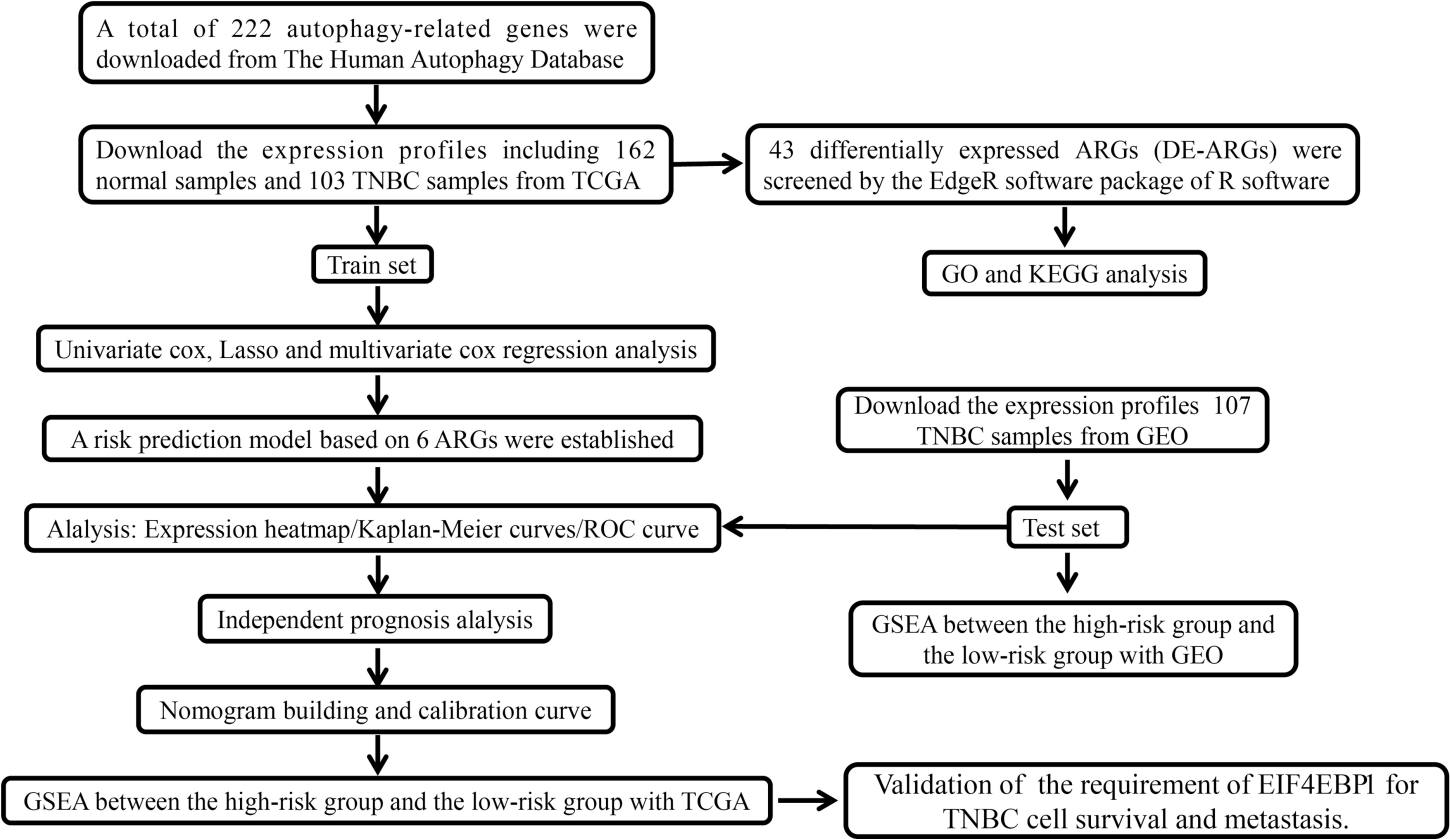
The flowchart describing the experimental design to establish and validate the prognostic signature in the study.

**Figure 2:**
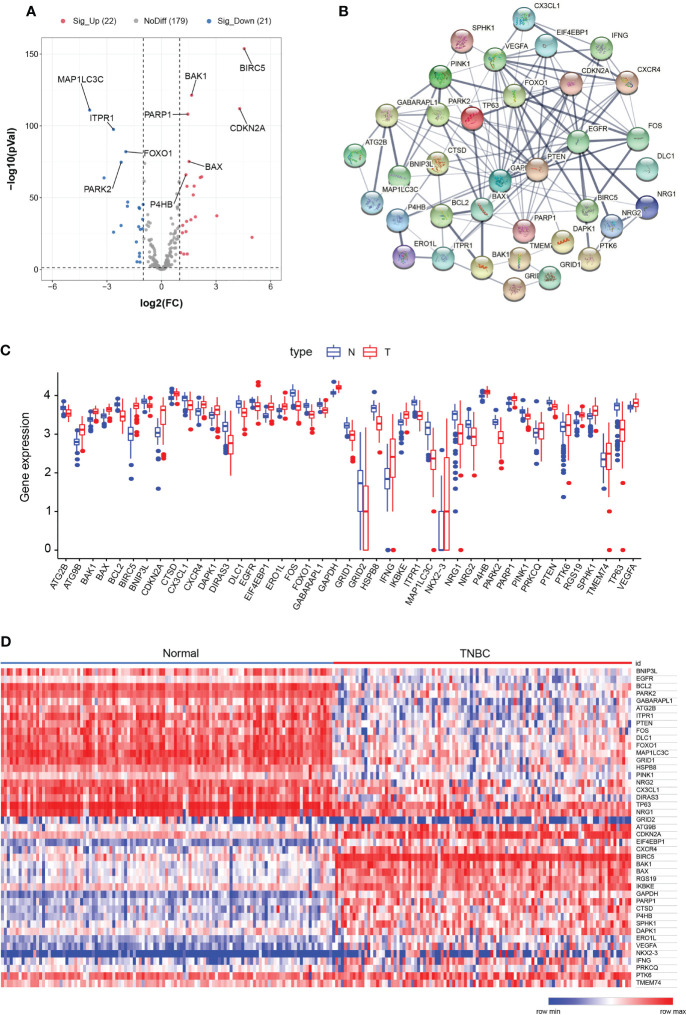
Differentially expressed autophagy-related genes. **(A)** Volcano map showed differentially expressed genes between normal samples and TNBC samples. Red dots represent significantly upregulated genes, blue dots represent significantly downregulated genes, and gray dots represent ARGs with no difference. **(B)** Protein–protein interactions (PPI) network of ARGs using the STRING database. **(C)** Box plots showed gene expression values of ARGs. Blue represents normal samples, and red represents tumor samples. **(D)** Heatmap showed the expression levels of ARGs. The color scale represented the expression levels normalized by z-score.

Autophagy has been reported to contribute to TNBC progression. In this study, we intend to construct the prognosis model using ARGs signature for TNBCs. The overall experimental design in this study is indicated as a diagram (**Figure 1**). We first obtained the expression profiles containing 162 normal samples and 103 TNBC samples from TCGA database and gathered 222 ARGs from the HADb database. A total of 43 differentially expressed ARGs (DE-ARGs) were identified by comparing normal and tumor samples with the cutoff criteria of FDR <0.05 and |log_2_FC| > 1. The volcano map (**Figure 2A**), box plots (**Figure 2C**), and heatmap (**Figure 2D**) demonstrated that 21 ARGs were significantly downregulated, while 22 ARGs were upregulated in TNBC patients. These DE-ARGs interacted with each other forming an autophagy network as measured by STRING (**Figure 2B**). Moreover, we observed that many mutations occur on these DE-ARGs in TNBCs (**Supplementary Figure 1**).

### Enrichment Analysis of DE-ARGs

To determine the functional enrichment of DE-ARGs, we performed GO and KEGG enrichment analysis. We found that these 43 DE-ARGs were highly correlated to autophagy, process utilizing autophagic mechanism, and peptidyl serine modification in the term of biological process (BP). In the aspect of cellular components (CCs), these genes were enriched in the nuclear envelope, mitochondrial outer membrane, and organelle outer membrane. For the molecular functions (MFs), these genes were mainly concentrated in ubiquitin protein ligase binding, ubiquitin-like protein ligase binding, and protein phosphatase (**Supplementary Figure 2A**). Moreover, KEGG enrichment analysis indicated that the DE-ARGs were involved in the signaling pathways such as autophagy animal (*Homo sapiens*), epidermal growth factor receptor (EGFR) tyrosine kinase inhibitor resistance and apoptosis (**Supplementary Figure 2B**). Overall, these data suggested that these ARGs play a role in other biological process in addition to autophagy.

### Construction of a Prognostic ARG Signature of TNBC in the Train Set

**Figure 3:**
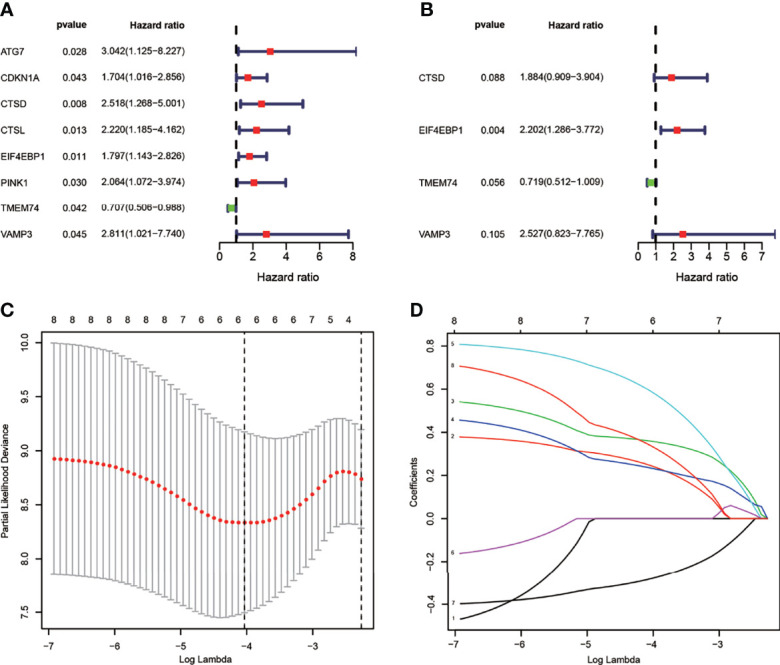
Identification of ARGs with prognostic value in breast cancer. **(A)** Univariate Cox regression hazard model for the overall survival in TNBC. **(B)** Multivariate Cox regression hazard model for the overall survival in TNBC. **(C)** LASSO regression analysis of ARGs based on univariate Cox regression analysis. The horizontal axis represents the log value of the independent variable λ, whilst the vertical axis represents the partial likelihood deviance of the log value of each independent variable λ. **(D)** Coefficients were calculated for each lambda. Each line represents a gene confidence value.

To build the ARG prognostic model, we first analyzed the risk score of all ARGs in TNBC by performing univariate Cox regression analysis. Eight ARGs were screened out including seven potential risky genes and one potential protective gene (**Figure 3A**). Subsequently, we performed LASSO regression analysis on the basis of univariate Cox regression analysis (**Figures 3C, D** **)**. Then, we constructed the prognostic ARG signature for OS using CDKN1A, CTSD, CTSL, EIF4EBP1, TMEM74, and VAMP3 by LASSO regression. Finally, we performed multivariate Cox regression analysis and screened out four ARGs including three potential risk genes and one potential protective gene (**Figure 3B**).

**Figure 4:**
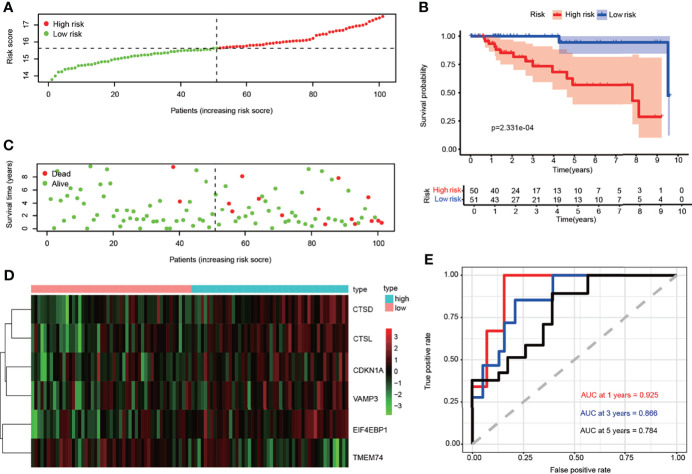
Correlation between the risk score and overall survival in the train set. **(A)** The risk score stratified the TNBC patients into high-risk groups (“High” red line) and low-risk groups (“Low” green line). **(B)** Kaplan–Meier survival curves to show survival probability comparing the high-risk groups with low-risk group. **(C)** Comparison of survival time and survival status of patients in TNBC between high- and low-risk groups. Green plots for alive, red plots for dead. **(D)** Heatmap showing expression of the six genes screened from ARGs in TNBC. Blue color represents high-risk group, while pink color represents low-risk group. The color scale represented the expression levels normalized by z-score. **(E)** Time-dependent ROC curves for survival prediction by the risk score.

Next, we tested if the expression of these six ARGs was correlated with the prognosis of TNBCs. We found that the high expression of EIF4EBP1 (p = 0.046), CTSL (p = 0.009), and CTSD (p = 0.07) might indicate a worse prognosis. There were no statistical differences in the survival analyses of CDKN1A (p = 0.362), TMEM74 (p = 0.107), and VAMP3 (p = 0.189) (**Supplementary Figure 3**). Then, we want to validate whether this ARG signature can predict the OS of TNBC. We first divided TNBC patients into “high risk” (n = 50) and “low risk” (n = 51) group according to the threshold of the median risk score (**Figure 4A**). The risk score for each patient was calculated based on the formula: risk score = (0.246026 × CDKN1A) + (0.359130 × CTSD) + (0.234375 × CTSL) + (0.590736 × EIF4EBP1) + (−0.281261 × TMEM74) + (0.338378 × VAMP3). Patients were assigned to high-risk (n = 50) and low-risk groups (n = 51) according to the threshold of the median risk score. Patients with higher scores were more likely to have a poorer prognosis (**Figure 4C**). A heatmap was used to visualize differences in expression levels of the six ARGs between groups (**Figure 4D**). Survival curves further indicated that patients in the high-risk group showed a significantly lower probability of survival compared to low-risk group (p < 0.05) (**Figure 4B**). ROC analysis revealed that the area under the curves (AUCs) for 1-, 3-, and 5-year OS were 0.925, 0.866, and 0.784, respectively (**Figure 4E**). Principal component analysis indicated that the distribution patterns of high- and low-risk populations were different based on the train set (**Supplementary Figure 6A**). These data suggested that ARGs signature in our model could benefit the prognosis prediction of TNBCs.

### Validation of the Risk Score of ARG Signature in a GEO Test Set

**Figure 5:**
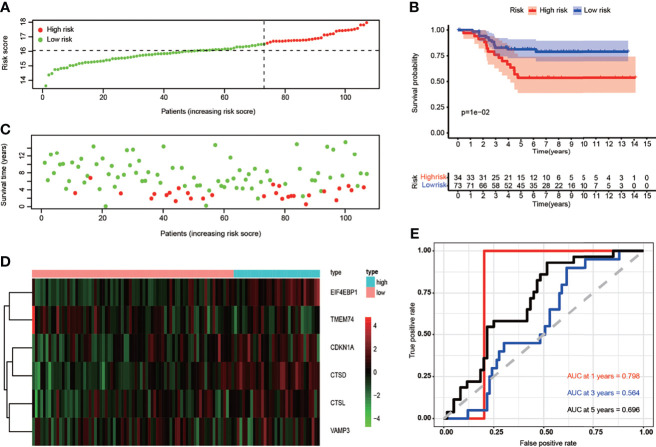
Correlation between the risk score and overall survival in the test set. **(A)** The risk score stratified the TNBC patients into high-risk groups (“High” red line) and low-risk groups (“Low” green line). **(B)** Kaplan–Meier survival curves to show survival probability comparing the high-risk groups with low-risk group. **(C)** Survival time and survival status of patients in TNBC comparing high-risk group with low-risk group. Green plots for alive, red plots for dead. **(D)** Heatmap showing expression of the six genes screened from ARGs in TNBC. Blue color represents high group, while pink color represents low group. The color scale represented the expression levels normalized by z-score. **(E)** Time-dependent ROC curves for survival prediction by the risk score.

To further validate the prognostic and predictive role of ARGs signature, we employed another GEO cohort as a test set to calculate risk scores using the same formula used in the train set. The patients from the test set were divided into the high-risk group (n = 34) and low-risk group (n = 73) by the median value of the train set (**Figure 5A**), and the higher risk score predicted poorer prognosis in the patients (**Figure 5C**). A heatmap was presented to visualize the different expression levels of the six ARGs between test groups (**Figure 5D**). Similar to the train set, patients in the high-risk score group showed a poorer prognosis compared to the low-risk group in the test set (p < 0.05) (**Figure 5B**). Time-dependent ROC analysis showed that the prognostic accuracy of OS was 0.798 at 1 year, 0.564 at 3 years, and 0.696 at 5 years (**Figure 5E**). Principal component analysis indicated that the distribution patterns of high- and low-risk populations were different based on the test set (**Supplementary Figure 6B**).

### Independent Prognostic Indicator of the Prognostic Risk Model

**Figure 6:**
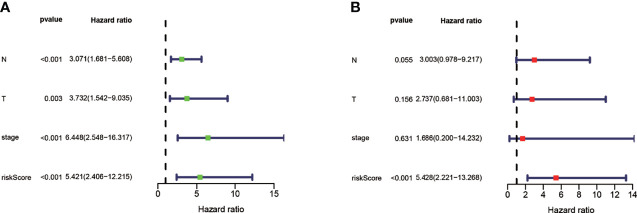
Analysis of the risk scores as an independent prognostic indicator. **(A)** Univariate Cox regression analysis identified that N, T, stage, and risk score were significantly associated with OS prediction. **(B)** Multivariate Cox regression analysis identified that risk score was independent prognostic factor for TNBC.

To confirm whether risk scores can be used as an independent predictor for TNBC patients’ survival, we further performed univariate analysis in the training set. Univariate Cox regression analysis revealed that N, T, stage, and risk score were meaningful for predicting OS (**Figure 6A**). Subsequently, we performed a multivariate Cox regression analysis to verify risk score as an independent predictor (p < 0.001) (**Figure 6B**). Moreover, we identified that the expression of CTSD was significantly associated with stages (p = 0.025) (**Supplementary Figure 4A**) and T (p = 0.031) (**Supplementary Figure 4B**). These data demonstrated that our model could be a reliable prognostic predictor and biomarker in addition to known clinical classification.

### Construction of the Nomogram and Performance Validation

**Figure 7:**
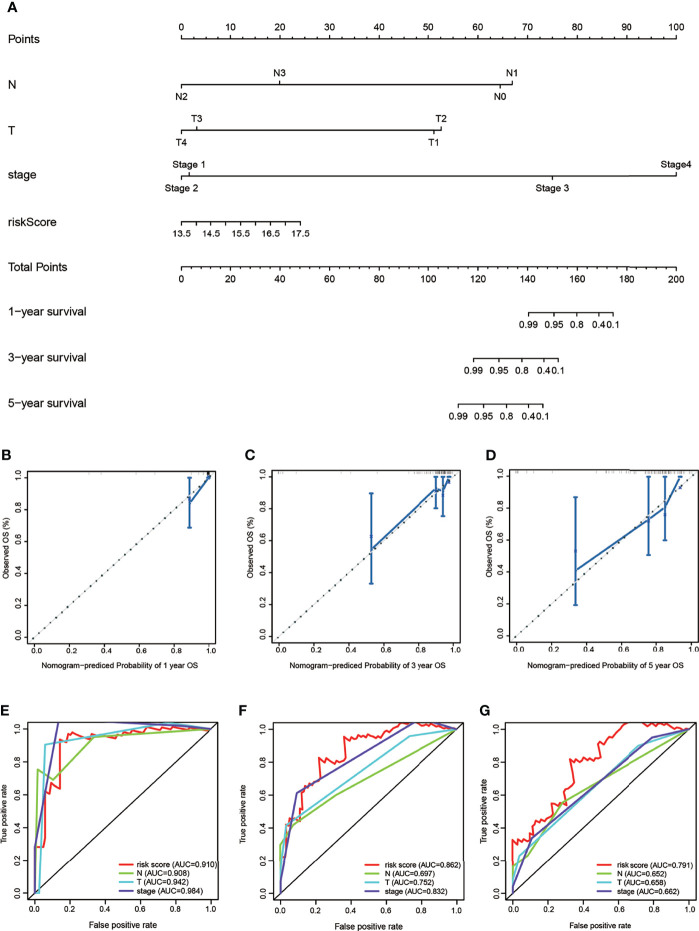
The nomogram to predict overall survival of TNBC patients of TAGC cohort. **(A)** The nomogram for predicting survival proportion of patients in 1, 3-, and 5 years. **(B–D)** The calibration plots for predicting patient survival at 1, 3, and 5 years. **(E–G)** The ROC curve of OS for risk score, N, T, and stage at 1, 3, and 5 years.

To provide the clinician with a better quantitative method to predict prognosis of TNBC patient, we established a nomogram with multiple factors including N, T, stage, and risk score (**Figure 7A**). The nomogram was used to evaluate the survival probability of 1, 3, and 5 years. Nomograms showed a good performance with a high C-index of 0.764, suggesting that it could be served as an effective tool for the prognostic evaluation of patients with TNBC. In addition, we constructed calibration curves, which showed that the predicted and actual survival rates were in agreement with 1, 3, and 5 years (**Figures 7B–D**). Finally, we compared the predictive accuracy for TNBC between the nomogram and clinicopathological risk factors by the values of AUC. Our model’s AUC value (AUC of 1-, 3-, and 5-year OS) was higher than the traditional prognostic scoring systems (**Figures 7E–G**). These findings revealed that the nomogram with our risk scores can precisely evaluate the OS in patients with TNBC.

### Enrichment Analysis Between High- and Low-Risk Group

Finally, we performed GSEA between the high- and the low-risk group in TCGA and GSE58812 cohort, respectively, to further provide biological insight. We found that the enriched KEGG pathways of the high-risk group in TCGA cohort included apoptosis, Fc epsilon RI signaling pathway, glycosylphosphatidylinositol GPI anchor biosynthesis, lysosome, and olfactory transduction. Meanwhile, enriched KEGG pathways of low-risk group included protein export, RIG-I like receptor signaling pathway, RNA polymerase, taste transduction, and Toll-like receptor signaling pathway (**Supplementary Figure 5A**). In addition, KEGG enrichment pathway analysis of high-risk group in GSE58812 cohort indicated that the genes were enriched in ABC transporters, arginine and proline metabolism, lysosome, pathogenic *Escherichia coli* infection, and pentose phosphate pathway. KEGG enrichment pathways analysis of the low-risk group in GSE58812 cohort were mainly concentrated in the regulation of autophagy, RIG-I-like receptor signaling pathway, RNA degradation, spliceosome, and *Vibrio cholerae* infection (**Supplementary Figure 5B**).

### Knockdown of EIF4EBP1 Inhibited TNBC Cell Proliferation and Migration

**Figure 8:**
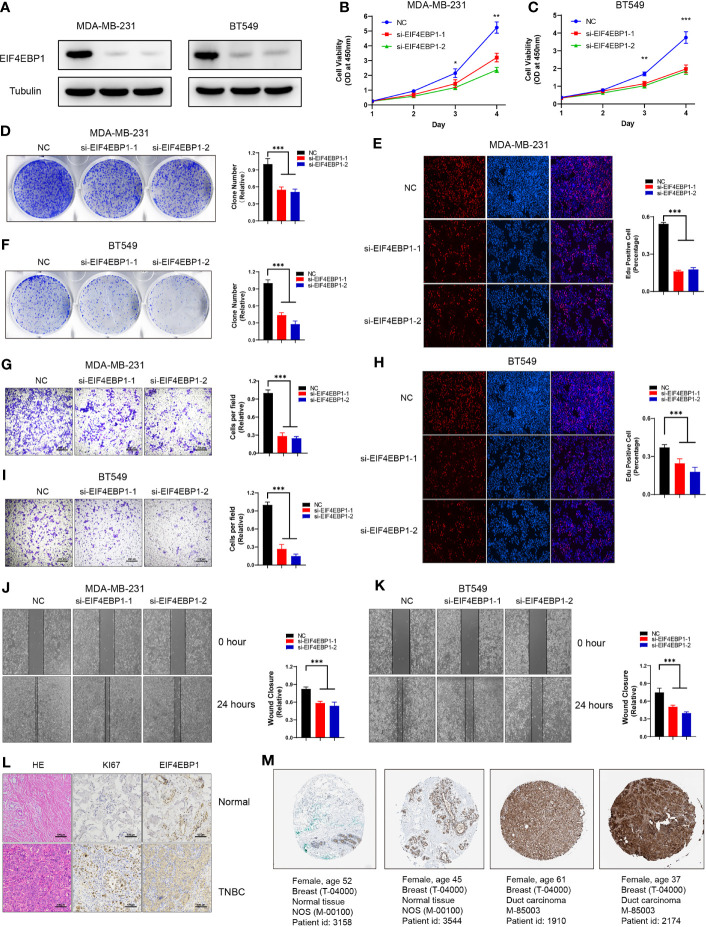
EIF4EBP1 is required for TNBC cell survival and migration. **(A)** Western blot to show knockdown efficiency of EIF4EBP1 in MDA-MB-231 and BT549 cells by two independent siRNAs. **(B, C)** Cell proliferation of MDA-MB-231 cells **(B)** or BT549 cells **(C)** transfected with control or EIF4EBP1 siRNAs was measured by CCK8. **(D, F)** Colony formation of MDA-MB-231 cells or BT549 cells transfected with control or EIF4EBP1 siRNAs was measured by ImageJ. **(G, I)** Transwell assay to show the cell metastasis of control cells compared to EIF4EBP1 knockdown cells. **(E, H)** Edu assay to show the cell proliferation of control cells comparing to EIF4EBP1 knockdown cells. **(J, K)** Wound healing assay to show the cell migration of control cells compared to EIF4EBP1-depleted cells. **(L)** Representative images of HE staining, immunostaining of KI67, and EIF4EBP1 in primary TNBC samples versus normal samples. **(M)** Representative images of immunostaining of EIF4EBP1 in primary TNBC samples compared to normal tissues from the HPA database. All data are shown as mean ± SEM. *p < 0.05, **p < 0.01, ***p < 0.001 by one-way ANOVA.

We next want to test the biological function of these ARGs in our model in TNBC. Among these six genes, the function of EIF4EBP1 in TNBC remains unknown. We knocked down EIF4EBP1 using two independent siRNAs in two TNBC cell lines: MDA-MB-231 and BT549 (**Figure 8A**). Knockdown of EIF4EBP1 resulted in a dramatic decrease in cell growth and colony formation (**Figures 8B–D, F** **)**. Edu staining showed that knockdown of eIF4EBP induced a significant decrease in proliferation (**Figures 8E, H** **)**. In addition, EIF4EBP1 knockdown significantly impaired cell metastasis as measured by Transwell and wound healing assay (**Figures 8G, I–K**). Furthermore, we observed increased EIF4EBP1 expression in primary TNBC samples compared to adjacent normal tissues in collected three TNBC patients (**Figure 8L**). Based on the Human Protein Atlas database, the protein expression levels of EIF4EBP1 were evaluated by the CAB005032 antibody. Among 12 TNBC tissues examined, 6 cases had medium to high staining (4 medium and 8 high), while no cases had low staining. Representative immunohistochemistry (IHC) image showed that EIF4EBP1 staining was higher in TNBC than in normal tissues (**Figure 8M**). Overall, these findings suggest a potential oncogenic role of EIF4EBP1 in TNBCs supporting the importance of our prognosis model in TNBCs.

## Discussion

TNBC is one of the most served malignant tumors among women in the world. Although <20% of all diagnosed breast cancer patients are triple-negative breast cancer, there are still 25%–40% of patients of the total breast cancer population with metastases, accounting for a disproportionate number of deaths from breast cancer (13). Due to the lack of targetable receptors, TNBC represents a clinically challenging endeavor. Currently, treating TNBC mainly includes adjuvant chemotherapy plus surgical resection for an early stage and adjuvant chemotherapy for an advanced stage. However, surgical resection may provide an unsatisfactory effect because of its highly invasive growth pattern and developed metastasis. Additionally, chemotherapy effects are diminished due to tumor heterogeneity. Even worse, TNBC is insensitive to the usual hormone therapies because of lack of hormone receptors expression. Therefore, it is essential to establish a novel biomarker to predict the prognosis and provide reliable treatment targets of TNBC.

Autophagy is a self-degradative process that is important for balancing sources of energy at critical times in the development and in response to cellular stress, which plays a dynamic tumor-suppressive or tumor-promoting role in different contexts and stages of cancer development (14). Expression of Beclin1 and LC3, key regulators of autophagy, are higher in TNBC cells compared to the other breast cancer subtypes, with the lowest expression in the stroma of TNBC (8). High LC3B expression is not only associated with lymph node and distant metastasis but also correlated with shorter survival in patients with triple-negative breast carcinoma (9). Moreover, knockdown of autophagy-related genes (LC3 and Beclin1) inhibits autophagy and significantly suppresses cell proliferation, colony formation, migration, and induced apoptosis in MDA-MB-231 and BT-549 TNBC cells (11). Similarly, silencing of ATG5, ATG7, and Beclin1 reduces the proliferation of different TNBC cell lines (15). These data strongly suggest that autophagy is essential to the survival of TNBC cells, indicating that therapeutic targeting of autophagy genes may be a potential therapeutic strategy for TNBC in breast cancer.

Recently, several prognostic factors have been identified in previous research with the aim of helping decision-making in pursuit of tailored individual care for TNBC patients. Yiduo Liu et al. screened four heterogeneous-related genes (FAM83B, KITLG, RBM24, and S100B) from 105 genes to construct a prognostic signature in the disease-free interval (DFI) of TNBC (16). Chao Li et al. identified a prognosis-related signature associated with energy metabolism including eight energy metabolism-associated genes (IL1RL2, FBLN7, CA3, PDE1B, SLURP1, CILP, AQP7, and TPSB) in triple-negative breast cancer (17). Ji Yeon Kim et al. obtained 13 immune-related genes to predict distant recurrence of early TNBC (18). Huan-Ming Hsu et al. unveiled six immunoglobulin genes as biomarkers in TNBC patients and explored the potential biomarkers of recurrence for TNBC (19). Fei Chen et al. identified nine steroid hormone-related genes as independent prognostic markers based on RNA-seq analysis in TNBC (20). To some degree, these models all showed better predicting ability than other clinicopathological factors and added prognostic value to the TNM staging system. Many studies have shown that autophagy plays an important role in prognosis of multiple cancers. However, to our knowledge, autophagy-related prognostic risk models have not been established for TNBC yet. It is of great significance to develop an autophagy-associated biomarker for TNBC prognosis prediction. In this study, we proposed that the prognostic risk model based on ARGs provided good prediction of prognosis for patients with TNBC, which may help clinical decision-making in pursuit of individual patient care.

In this study, we mined 43 DE-ARGs by comparing TNBC samples to normal samples. Subsequently, GO and KEGG pathways enrichment of these DE-ARGs revealed that some cancer-related signaling pathways were significantly enriched, such as autophagy, apoptosis, and HIF-1 signaling pathway. Through further univariate Cox regression and LASSO regression analysis, six ARGs (CDKN1A, CTSD, CTSL, EIF4EBP1, TMEM74, and VAMP3) were obtained. Finally, we established a prognostic signature based on the six ARGs to effectively predict the prognosis of TNBC patients.

Consistent with earlier research, these six ARGs have been reported to play multiple roles in various cancer types. A wide array of studies documented that CTSD promoted tumor growth, invasion, and metastatic dissemination in breast cancer (21–24). Wei Zhang et al. found that the CTSL expression levels in malignant ovarian tumors were significantly higher than in normal or benign tissues (25). Furthermore, Luosheng Zhang et al. also suggested that CTSL is involved in the proliferation and invasion of ovarian cancer cells (26). Some studies indicated that EIF4EBP1 is involved in the progression of various cancer types (including renal cell carcinoma, breast cancer) through regulating the transcription level of BRDT (27, 28). Kevin Luftman et al. investigated the function of VAMP3, and they found that silencing of VAMP3 could inhibit cancer metastasis (29). These results were consistent with our findings. Of note, data on the prognostic relevance of CDKN1A expression showed that increased expression of CDKN1A were associated with poor prognosis in esophageal, ovarian, prostate cancers, and gliomas (30–36). In contrast, some studies also indicated that low expression level of CDKN1A was correlated with better survival in cervical, gastric, cholangiocarcinoma, and ovarian cancers (37–39). These findings suggested the dual role of CDKN1A in cancer, which needs to be further explored. Interestingly, TMEM74 had been regarded as an oncogene in various cancers including liver cancer, lung cancer, breast cancer, colon cancer, cervical cancer, and hepatic carcinoma. Higher expression level of TMEM74 was associated with poorer survival, which was not consistent with our study (40). This could be due to the variation in genetic context in different cancer types. The high expression level of TMEM74 might play a protective role in TNBC, not in others.

This study needs to be expanded in the future, as the sample number of each cohort used is relatively small. Additionally, further studies are required to understand the role of ARGs in TNBC and its potential molecular mechanisms.

## Conclusions

Based on six ARGs (CDKN1A, CTSD, CTSL, EIF4EBP1, TMEM74, and VAMP3), we developed a risk prediction model that can help clinical doctors effectively predict the survival status of TNBC patients. Our data suggested that EIF4EBP1 might promote the proliferation and migration in TNBC cell lines. These findings provided a novel insight into the vital role of the autophagy-related genes in TNBC and may provide new therapeutic targets for TNBC.

## Data Availability Statement

The original contributions presented in the study are included in the article/**Supplementary Material**. Further inquiries can be directed to the corresponding author.

## Ethics Statement

The studies involving human participants were reviewed and approved by ethics committee of Xinxiang University. The patients/participants provided their written informed consent to participate in this study. Written informed consent was obtained from the individual(s) for the publication of any potentially identifiable images or data included in this article.

## Author Contributions

RJ designed the research and wrote the paper. CY and QL downloaded and analyzed the data. CY conducted the cell-culture-related experiment. All authors have agreed to the manuscript. All authors contributed to the article and approved the submitted version.

## Funding

This work was supported by the Key Scientific and Technological Project of Henan Province (Grant No. 202102310416) and the Key Scientific Research Projects of Henan Colleges and Universities (Grant No. 21A310008).

## Conflict of Interest

The authors declare that the research was conducted in the absence of any commercial or financial relationships that could be construed as a potential conflict of interest.

## Publisher’s Note

All claims expressed in this article are solely those of the authors and do not necessarily represent those of their affiliated organizations, or those of the publisher, the editors and the reviewers. Any product that may be evaluated in this article, or claim that may be made by its manufacturer, is not guaranteed or endorsed by the publisher.

## Supplementary Material

Supplementary Figure 1 Mutation analysis of ARGs in TNBC in the TCGA database.

Supplementary Figure 2 GO and KEGG enrichment analysis of DE-ARGs in TNBC. **(A)** DE-ARGs GO enrichment analysis. BP stands for biological process, CC stands for cellular component, MF stands for molecular function. **(B)** DE-ARGs KEGG enrichment analysis.

Supplementary Figure 3 Kaplan-Meier curves of the selected six ARGs in the TCGA database were plotted.

Supplementary Figure 4 The correlation between CTSD expression and clinicopathological variables. **(A)** The expression level of CTSD was reversely related to stage in TNBC. **(B)** The expression level of CTSD was reversely related to T stage in TNBC.

Supplementary Figure 5 Gene set enrichment analysis (GSEA) identifies KEGG pathways associated with high-risk groups and low-risk groups in train set **(A)** and test set **(B)**.

Supplementary Figure 6 Principal component analysis between high-risk groups and low-risk groups in train set **(A)** and test set **(B)**.
