# Supplementary figures and images for: Construction and Validation of a Prognostic Risk Model for Triple-Negative Breast Cancer Based on Autophagy-Related Genes
Source: Front Oncol. 2022 Feb 4;12:829045. doi: 10.3389/fonc.2022.829045 (PMC8854264; doi:10.3389/fonc.2022.829045)

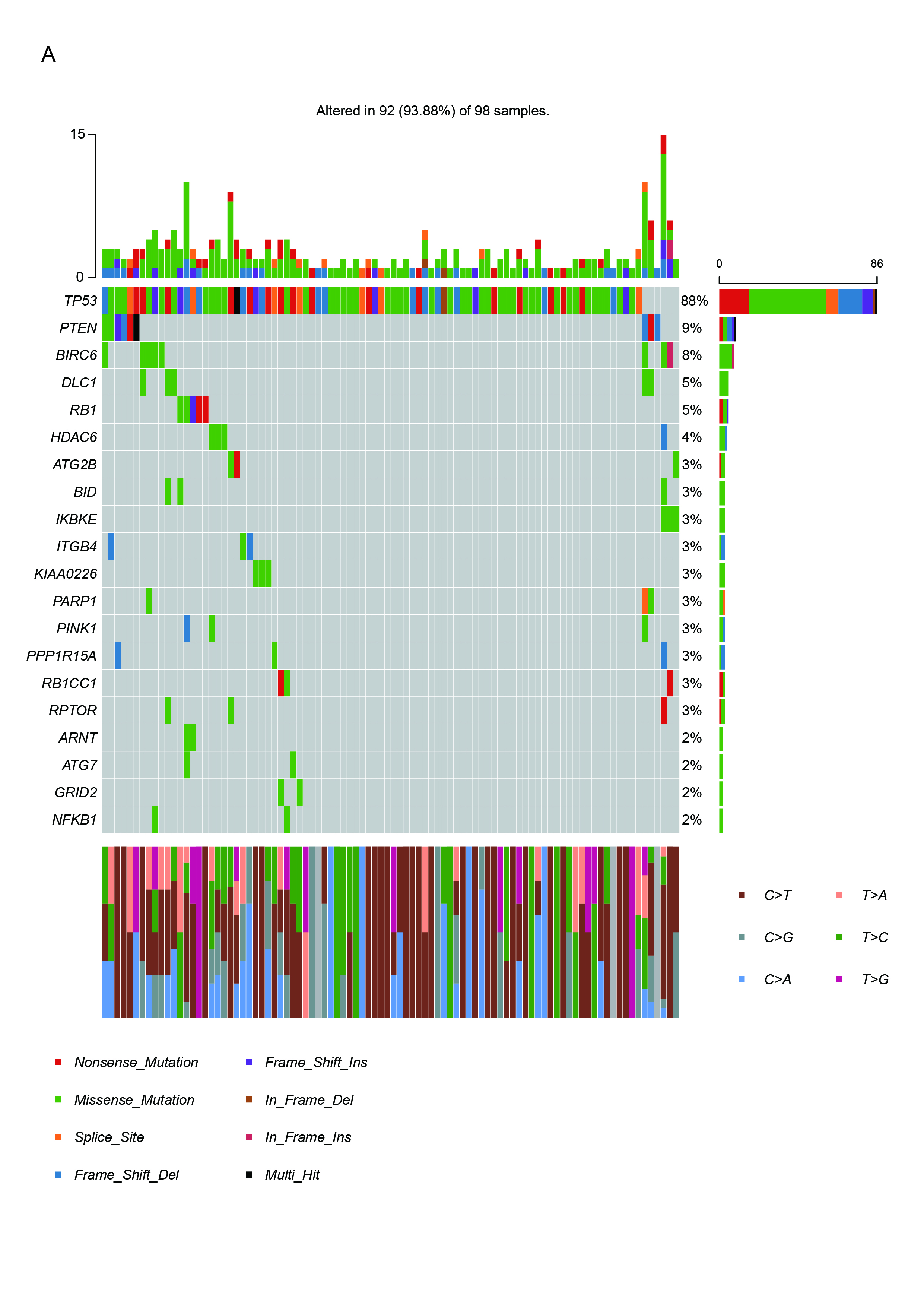

Supplement: Supplementary Figure 1 — Mutation analysis of ARGs in TNBC in the TCGA database. [file Image_1.jpg]

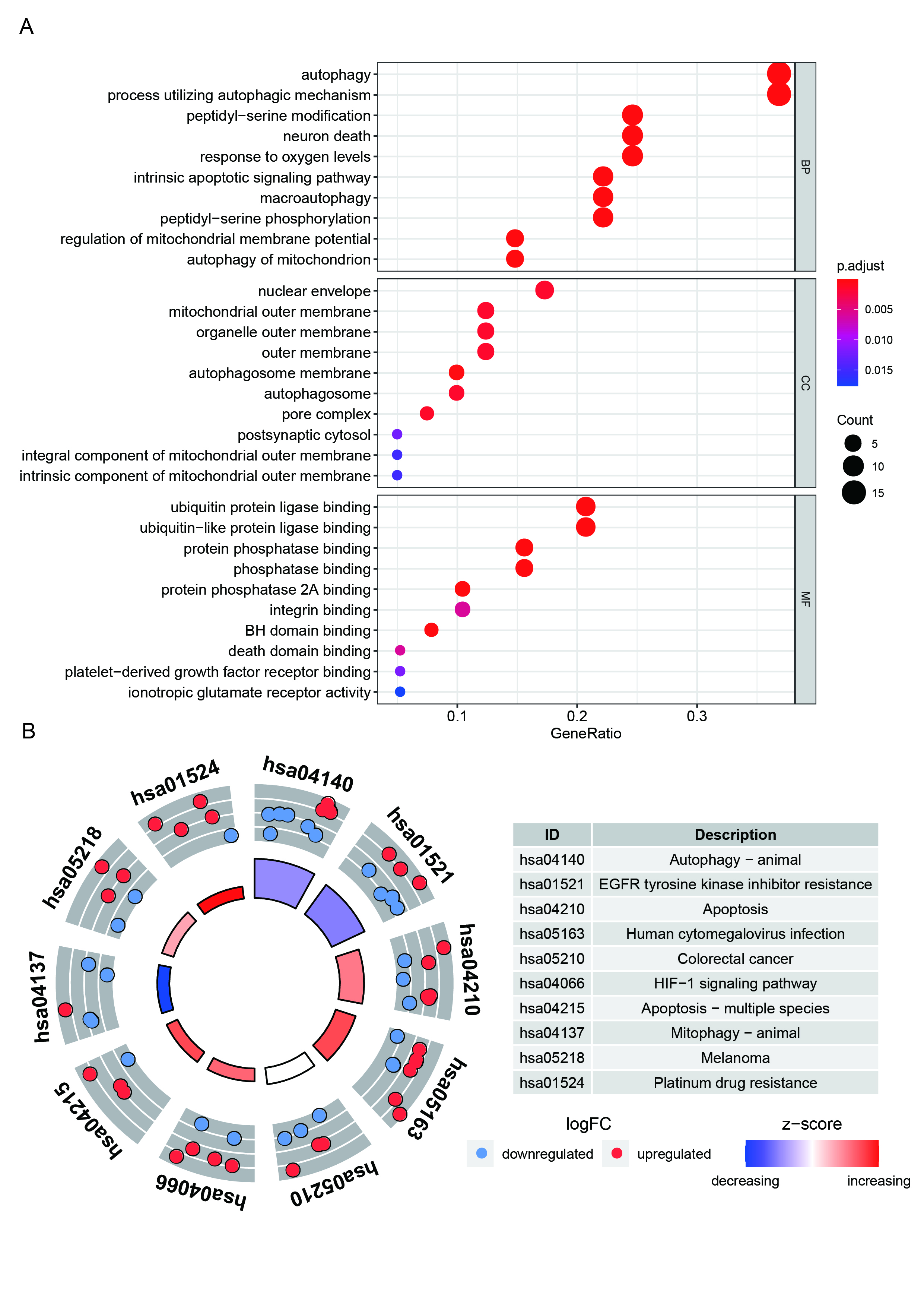

Supplement: Supplementary Figure 2 — GO and KEGG enrichment analysis of DE-ARGs in TNBC. (A) DE-ARGs GO enrichment analysis. BP stands for biological process, CC stands for cellular component, MF stands for molecular function. (B) DE-ARGs KEGG enrichment analysis. [file Image_2.jpg]

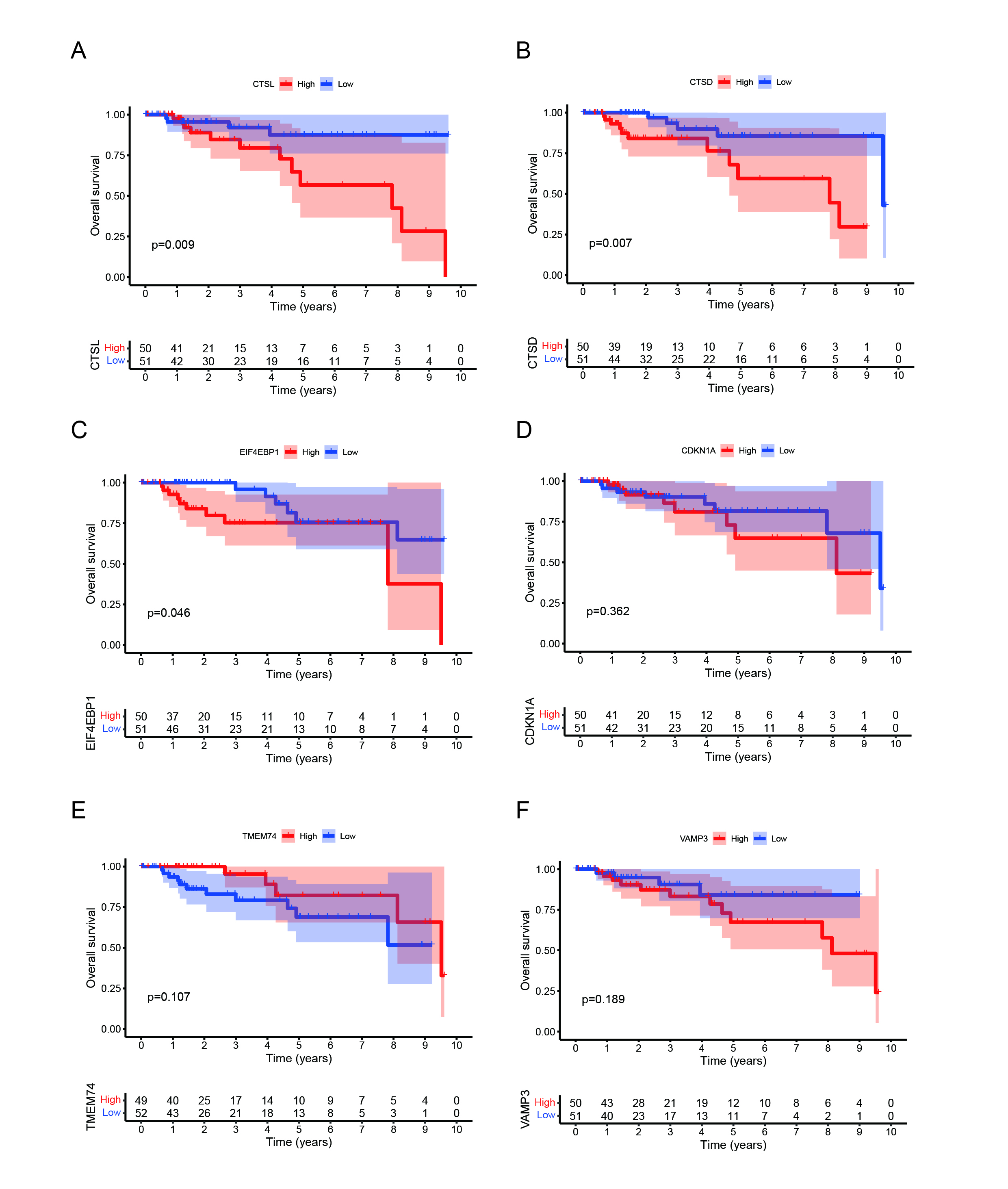

Supplement: Supplementary Figure 3 — Kaplan-Meier curves of the selected six ARGs in the TCGA database were plotted. [file Image_3.jpg]

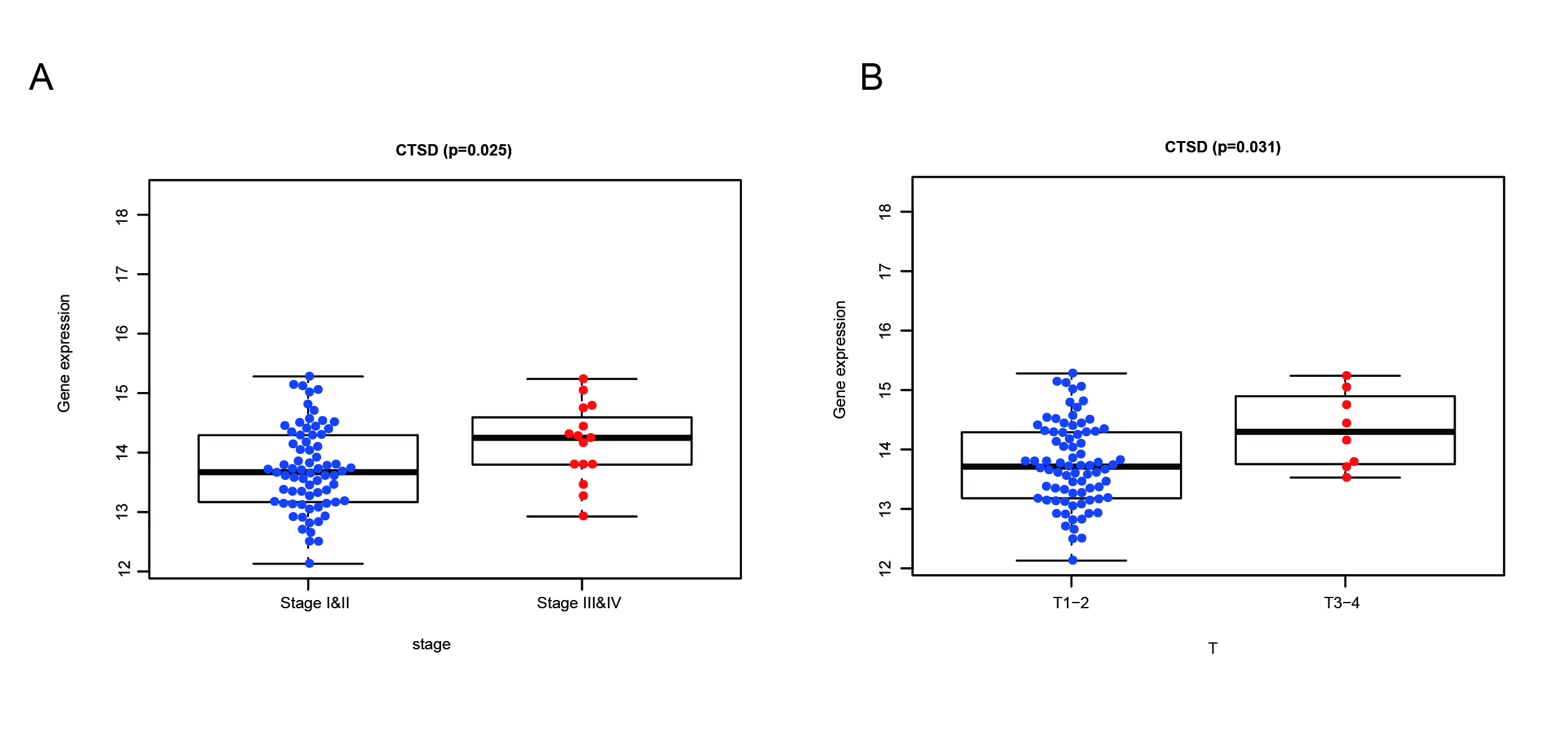

Supplement: Supplementary Figure 4 — The correlation between CTSD expression and clinicopathological variables. (A) The expression level of CTSD was reversely related to stage in TNBC. (B) The expression level of CTSD was reversely related to T stage in TNBC. [file Image_4.jpg]

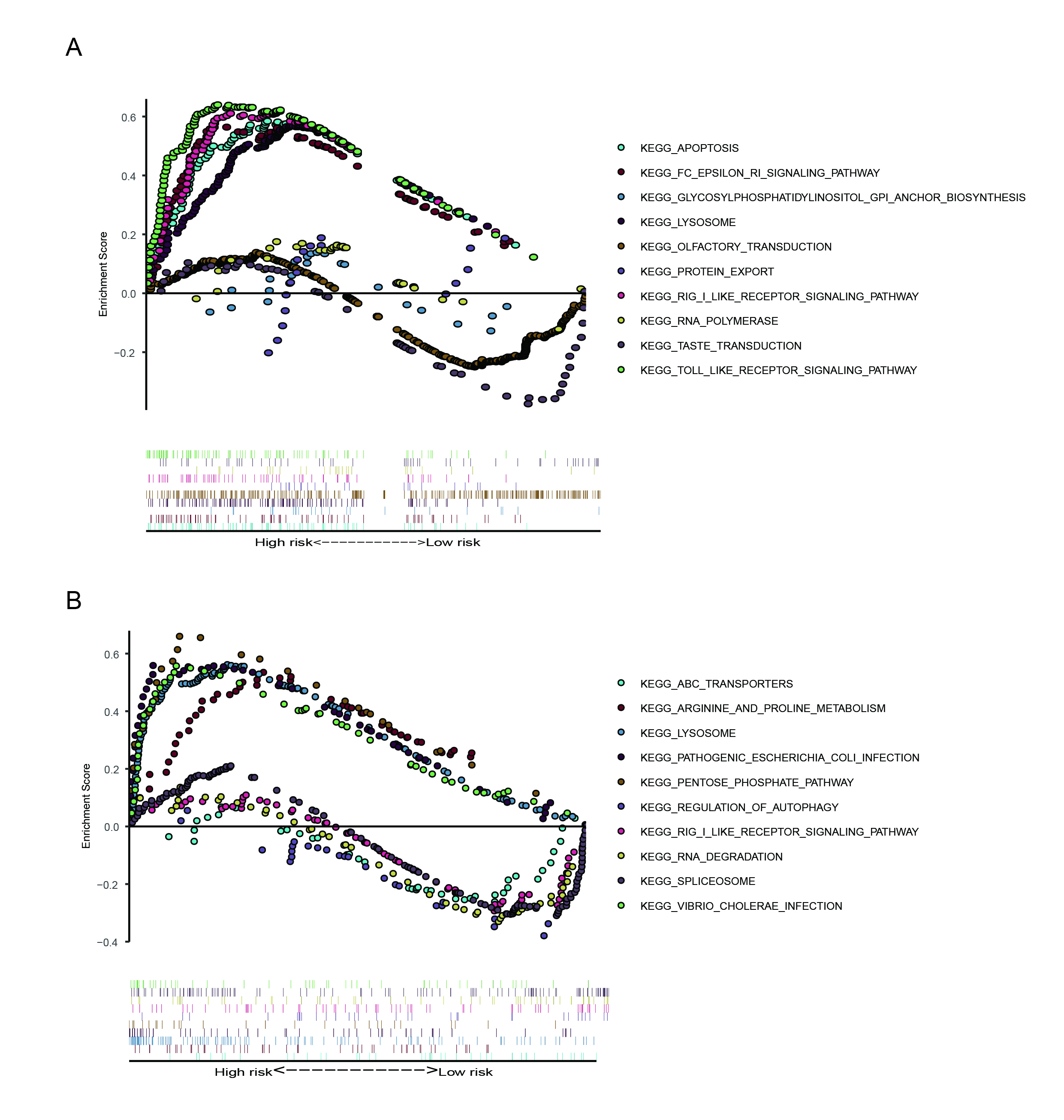

Supplement: Supplementary Figure 5 — Gene set enrichment analysis (GSEA) identifies KEGG pathways associated with high-risk groups and low-risk groups in train set (A) and test set (B). [file Image_5.jpg]

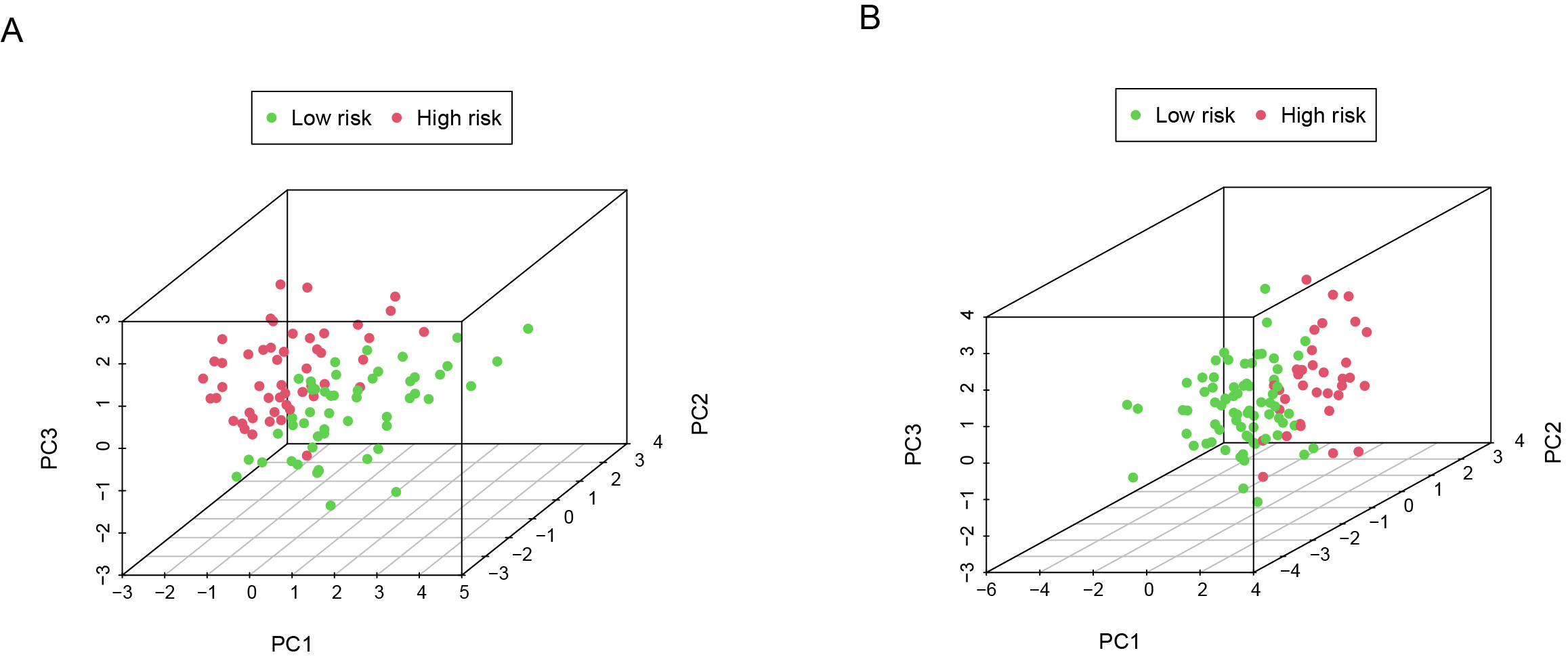

Supplement: Supplementary Figure 6 — Principal component analysis between high-risk groups and low-risk groups in train set (A) and test set (B). [file Image_6.jpg]
